# Structure and role of the linker domain of the iron surface-determinant protein IsdH in heme transportation in *Staphylococcus aureus*

**DOI:** 10.1016/j.jbc.2022.101995

**Published:** 2022-04-29

**Authors:** Sandra Valenciano-Bellido, Jose M.M. Caaveiro, Koldo Morante, Tatyana Sushko, Makoto Nakakido, Satoru Nagatoishi, Kouhei Tsumoto

**Affiliations:** 1 Department of Bioengineering, School of Engineering, The University of Tokyo, Tokyo, Japan; 2 Laboratory of Global Healthcare, Graduate School of Pharmaceutical Sciences, Kyushu University, Fukuoka, Japan; 3 Department of Chemistry and Biotechnology, Graduate School of Engineering, The University of Tokyo, Tokyo, Japan; 4 Institute of Medical Science, The University of Tokyo, Tokyo, Japan

**Keywords:** hemoglobin, heme, x-ray crystallography, hydrogen exchange mass spectrometry, *Staphylococcus aureus*, hemoglobin receptor, iron surface determinant system, heme acquisition, heme transport, structure-function, DMSO, dimethyl sulfoxide, DSC, differential scanning calorimetry, Hb, hemoglobin, HDX-MS, Hydrogen-Deuterium Exchange Mass Spectrometry, His_6_, hexahistidine tag, ITC, isothermal titration calorimetry, IMAC, immobilized-metal affinity chromatography, IPTG, isopropyl-β-D-thiogalactopyranoside, Isd, Iron surface determinant, NEAT, NEAr-iron transporter, PBS, Phosphate Buffer Saline, rHb, recombinant Hb, RMSD, root-mean-square deviation, SDS-PAGE, sodium dodecyl sulfate–polyacrylamide gel electrophoresis

## Abstract

*Staphylococcus aureus* is a major cause of deadly nosocomial infections, a severe problem fueled by the steady increase of resistant bacteria. The iron surface determinant (Isd) system is a family of proteins that acquire nutritional iron from the host organism, helping the bacterium to proliferate during infection, and therefore represents a promising antibacterial target. In particular, the surface protein IsdH captures hemoglobin (Hb) and acquires the heme moiety containing the iron atom. Structurally, IsdH comprises three distinctive NEAr-iron Transporter (NEAT) domains connected by linker domains. The objective of this study was to characterize the linker region between NEAT2 and NEAT3 from various biophysical viewpoints and thereby advance our understanding of its role in the molecular mechanism of heme extraction. We demonstrate the linker region contributes to the stability of the bound protein, likely influencing the flexibility and orientation of the NEAT3 domain in its interaction with Hb, but only exerts a modest contribution to the affinity of IsdH for heme. Based on these data, we suggest that the flexible nature of the linker facilitates the precise positioning of NEAT3 to acquire heme. In addition, we also found that residues His45 and His89 of Hb located in the heme transfer route toward IsdH do not play a critical role in the transfer rate-determining step. In conclusion, this study clarifies key elements of the mechanism of heme extraction of human Hb by IsdH, providing key insights into the Isd system and other protein systems containing NEAT domains.

In order to produce infection, pathogenic bacteria must survive in a nutrient-deprived environment and under siege from the host defense systems. Pathogens are equipped with various so-called virulence factors to help them to prosper in this harsh environment and to counteract the host defenses (1, 2, 3). In particular, iron is a critical metal that functions as an electron carrier and as a cofactor in a vast number of proteins and therefore an essential nutrient for living organisms including pathogenic bacteria. Since the concentration of free iron in the cell is extremely low, pathogenic bacteria face a formidable obstacle to their growth. To acquire iron, bacteria must extract it from iron-bound or heme-containing proteins from the host, such as hemoglobin (Hb), serum transferrin, or lactoferrin (4).

The capture of heme (iron protoporphyrin IX) from the host’s heme-carrying proteins, such as Hb, represents a dauting challenge to bacterial pathogens (5, 6). In gram-positive bacteria, the peptidoglycan cell wall constitutes an additional barrier that complicates the transport of heme, thus requiring several surface-exposed proteins anchored to the cell wall in addition to ABC transporters in the cell membrane (7, 8). The best-characterized gram-positive heme transport system is that of *Staphylococcus aureus* (9). As the infection by *S. aureus* progresses into the hemolytic stage, the release of Hb from erythrocytes into the blood is initiated by the action of bacterial cytotoxins α- and γ-hemolysin (10, 11, 12). Heme acquisition by *S. aureus* is carried out by the iron surface determinant (Isd) system, whose expression is induced under iron-depleting conditions. The Isd system comprises nine proteins (9, 13, 14). The surface proteins IsdH and IsdB directly extract heme from Hb and Hb–haptoglobin complexes (14, 15, 16). From that moment, there is a sequential transfer of heme from the surface proteins IsdH and IsdB through intermediate transporters IsdA and IsdC in the cell wall to the membrane anchored protein IsdE and membrane transporter IsdF, shuttling heme to the cytoplasm (12, 14, 15, 17). In addition to transport proteins, the *isd* gene cluster also encodes the heme-degrading IsdG and IsdI proteins, both cytoplasmic heme-oxygenases with a unique heme coordination system that catalyzes the release of the iron atom (18). The function of the membrane-anchored IsdD protein remains unknown.

The protein IsdH is one of only two proteins of the Isd system exposed on the surface of the cell wall, ready to acquire heme directly from Hb of the host. IsdH is a 101-kDa protein composed of three NEAr-iron Transporter (NEAT) domains connected by linker regions (Fig. 1). NEAT1 and NEAT2, as well as the linker regions, bind and destabilize Hb, resulting in the extraction of heme by NEAT3 (12, 16). Previous reports have deciphered the individual or combined structure of NEAT domains and linker regions (19, 20, 21, 22), as well as the dynamics of binding to Hb (23, 24) and heme extraction (12, 14, 25). IsdH has a stoichiometry of 4:1 with Hb as reported in the crystal structure of the complex between the two proteins (20). Since Hb is a tetramer, the binding stoichiometry of IsdH with respect to each subunit of Hb is 1:1. Similarly, structural studies have shown that the stoichiometry for IsdB is also 4:1 (1:1 with respect to each chain of Hb) (26).

**Figure 1:**
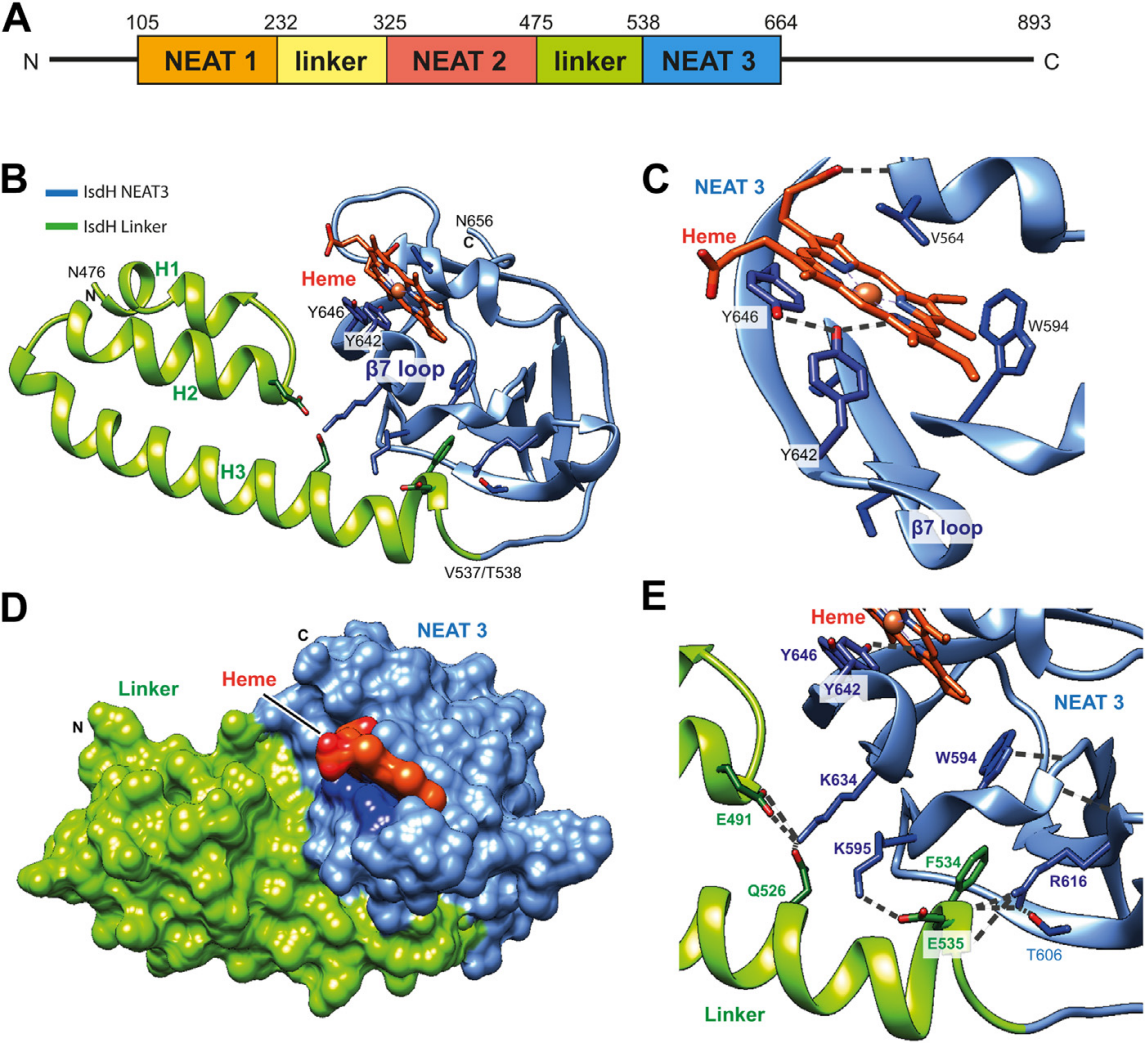
**Crystal structure of IsdH linker-NEAT3 in complex with Fe(III)-PPIX.***A,* modular structure of IsdH. *B*, overall structure of linker-NEAT3 at 1.8 Å resolution. The linker and NEAT3 domains are shown in *green* and blue, respectively. The heme group is depicted with *orange* sticks. The iron atom is shown as an *orange* sphere. Key residues for binding heme, Tyr642 and Tyr646, are also depicted with *blue* sticks. The identity of the first and the last residue of each domain is shown. *C*, close-up view of the heme-binding pocket in NEAT3. Some residues surrounding heme are depicted with sticks. *D*, surface representation using the same color scheme as above. *E*, close-up view of the interaction area between linker and NEAT3 domains. Relevant residues belonging to linker and NEAT3 domains are depicted with *green* and *blue* sticks, respectively. The figure was prepared using UCSF Chimera (53). NEAT, NEAr-iron Transporter.

Although a stronger interaction of IsdH with the α-chain has been described (19, 27), it was also determined that IsdH is capable of removing hemin from each subunit of Hb (23), and therefore, binding selectivity does not seem to affect heme acquisition. The linker domains have key roles in binding and destabilizing Hb (20, 23, 24, 28). Specifically, the interaction of the linker is essential for the distortion of the F-helix of the α-chain of Hb, weakening the interactions with heme in the binding pocket. The residue His89 of the F-helix is displaced during the destabilization process, and together with His45 from Hb, both have been proposed to be relevant during the heme extraction process (23, 24, 28). A similar mechanism has been proposed for the interaction between Hb and IsdB, including the distortion of F-helix caused by the linker domain connecting the NEAT1 and NEAT2 domains of IsdB (26, 29). Still, the overall role of the linker region and the contribution of the His residues of Hb in the heme extraction have not been experimentally addressed.

Herein we have focused on the linker-NEAT3 because of its importance to acquire the heme moiety. We aimed at advancing our basic understanding of the molecular mechanism of heme extraction and the role of the linker region. Based on structural models built from the new crystal structure of linker-NEAT3 with heme bound, reported here, and previous structural data, we also evaluated the influence of residues of Hb for the overall transfer rate. The collective picture that emerged from our structural, biophysical, and biochemical data indicated that the linker region plays a relevant role in the conformational dynamics and stability of IsdH but does not actively contribute to the binding affinity for heme. We propose a model of heme extraction in which the dynamic nature of the linker facilitates the precise positioning of NEAT3 to extract heme. Furthermore, we showed that residues His45 and His89 from Hb do not play a critical role for the rate of heme transfer. Our results further clarify the function of the linker for heme extraction and its role in the conformational dynamics and stability of IsdH.

## Results

### Crystal structure of IsdH linker-NEAT3 with heme bound

Previous studies reported the crystal structure of the NEAT3 domain of IsdH with heme bound (12) as well as the structure of domains NEAT2-linker-NEAT3 in complex with Hb (19, 20). Still, the structure of linker-NEAT3 with heme bound has not been characterized so far. Herein, the structure of linker-NEAT3 with heme bound was determined at a resolution of 1.8 Å (Fig. 1 and Table 1). We note that our attempt to crystallize linker-NEAT3 in the heme-free form resulted in crystals of only heme-free NEAT3 (without the linker domain). During the long crystallization experiment (weeks), the linker was cleaved at position Ala530 as inferred from the N-terminal sequence of crystal samples (see Experimental procedures and Tables S1–S3). The sequence of the cleaved linker-NEAT3 protein obtained from the crystals started with the sequence AVTEF, corresponding to residues Ala530 to Phe534. The sequence of freshly purified linker-NEAT3 was also determined, and it started with the expected sequence SNLQK, corresponding to residues N476 to K479. The first residue (serine) corresponded to a cloning artifact.

**Table 1:** Data collection and refinement statistics

Data collection	Linker-NEAT3 (heme)
Space group	P 2_1_ 2_1_ 2_1_
Unit cell	
a, b, c (Å)	49.9, 95.3, 99.4
α, β, γ (°)	90.0, 90.0, 90.0
Resolution (Å)	40.4–1.80
Wavelength	1.0000
Reflections (all)	246,137 (28,302)
Reflections (unique)	43,510 (6048)
*R*_*merge.*_	0.082 (0.454)
*R*_*p.i.m.*_	0.036 (0.217)
CC_1/2_	0.996 (0.865)
*I/σ (I)*	12.3 (2.8)
Multiplicity	5.7 (4.7)
Completeness (%)	97.6 (94.8)
**Refinement statistics**	
Resolution (Å)	40.4–1.80
*R*_*work*_/*R*_*free*_ (%)	18.5/22.6
No. protein chains	2
No. atoms	
Protein	3005
Heme	86
Water	381
B-factor (Å^2^)	
Protein	31.6
Heme	23.7
Water	33.0
Ramachandran plot	
Preferred (%)	92.5
Allowed (%)	7.5
Outliers (%)	0
RMSD bond (Å)	0.016
RMSD angle (°)	2.0
PDB	7W81

Statistical values given in parenthesis refer to the highest resolution bin.

The visible residues in the structure of linker-NEAT3 with heme bound comprised residues Asn476 to Val537 of the linker and residues Thr538 to Asn656 of NEAT3, in addition to the heme moiety (Fig. 1). The linker is composed of three α-helices (H1, H2, and H3) connected by short turns, whereas the NEAT3 domain is mostly made of β-strands with just two short helical structures. Overall, this structure was nearly indistinguishable to that of IsdH when bound to Hb, except for the presence of the heme molecule bound to NEAT3. The overall root-mean-square deviation (RMSD) of the structure determined herein with respect to that bound to Hb was 0.90 ± 0.06 Å, whereas the RMSD resulting from the comparison of the individual linker domains was 0.72 ± 0.05 Å, and that between NEAT3 domains was 0.80 ± 0.02 Å (19) (Fig. S1). The RMSD between the NEAT3 domain reported herein and isolated NEAT3 domain with heme bound (12) was even smaller (0.34 ± 0.01 Å) (Figs. 1 and S1). The iron-coordinating Tyr642 is located within a coordinating distance from the metal ion (2.11 ± 0.05 Å) in agreement with previous studies (12, 14, 22).

It is observed that the linker domain does not engage in direct contact with the heme moiety (Fig. 1). The linker domain interacts with the NEAT3 domain through the heme-binding pocket. An analysis of the linker-NEAT3 interface using the Protein Interfaces, Surfaces and Assemblies (PISA) service at the European Bioinformatics Institute (30) revealed that the linker domain is held together as a compact unit by a combination of interactions, such as nonpolar contacts, hydrogen bonds, and salt bridges. Collectively, these data suggest that the binding of heme to linker-NEAT3 does not result in conformational changes with respect to the Hb-bound form. The comparison of the structure of linker-NEAT3 bound to Hb (no heme bound) with the structure of linker-NEAT3 (with heme bound) suggests that binding of heme to IsdH would not cause substantial conformational changes in the linker region compared to that in the Hb-bound form. However, the conformation of the linker-NEAT3 unit before approaching Hb (*i.e.*, before heme is bound) is unknown. The excision of the linker from the NEAT domain in the absence of heme adds to the interest in resolving this question. To evaluate the dynamics and flexibility of the linker-NEAT construct in the absence and in the presence of heme, hydrogen-deuterium exchange mass spectrometry (HDX-MS) was performed as described in Experimental procedures.

### HDX-MS comparison of heme-bound and heme-free forms of linker-NEAT3

**Figure 2:**
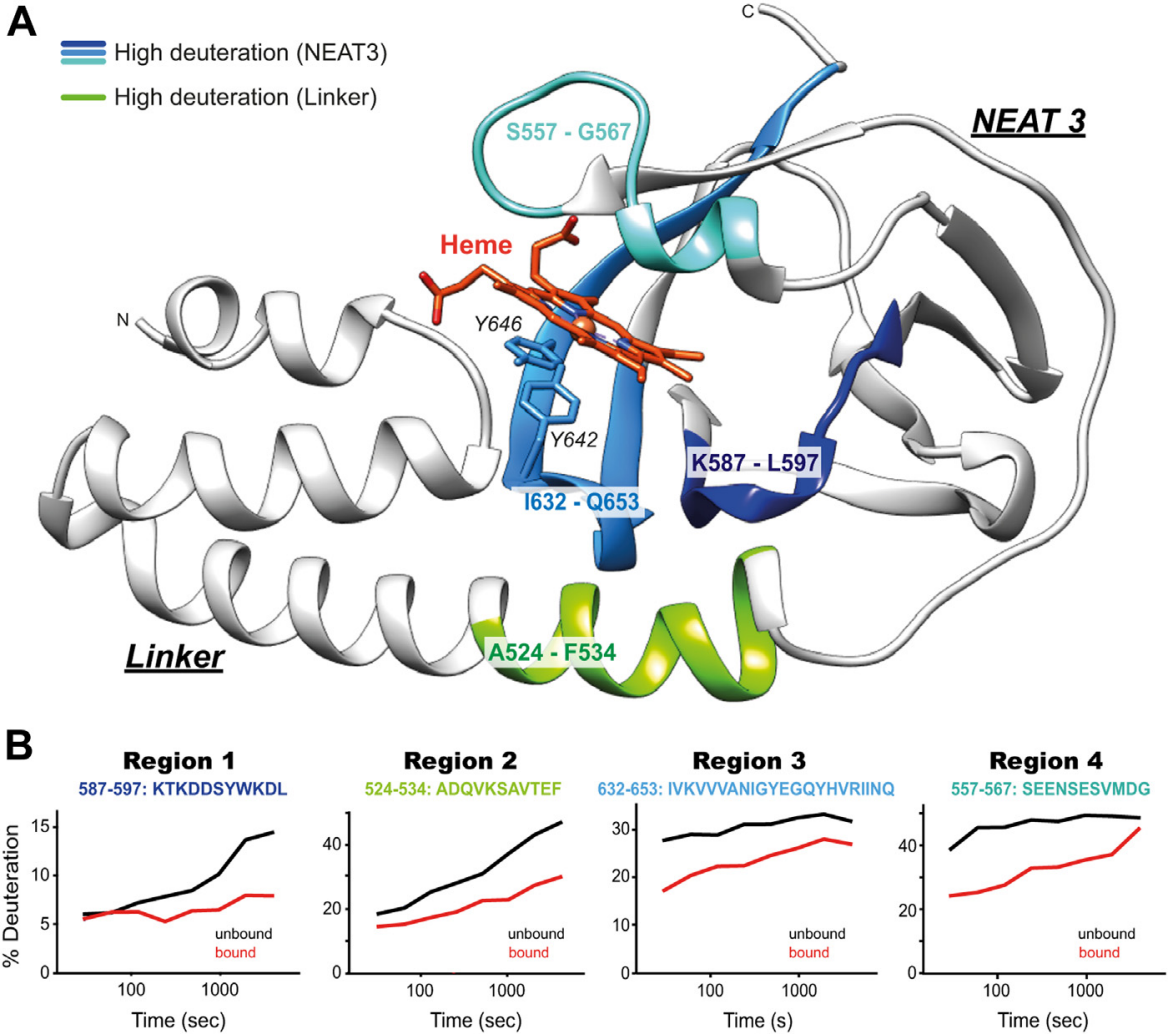
**Mapping flexibility of linker-IsdH by HDX-MS.***A*, four segments of interest are highlighted on the structure of IsdH linker-NEAT3 in complex with Fe(III)-PPIX. The flexible and/or exposed areas are highlighted, in *green* for the linker domain and in *blue* for the NEAT3 domain. The heme moiety is showed in *orange*. *B*, time course of deuterium exchange for the four regions mapped above. Each panel shows the degree of deuterium exchange for each segment (indicated at the *top* of each panel) in the heme-free (*black line*) and heme-bound (*red line*) forms. HDX-MS, hydrogen-deuterium exchange mass spectrometry; NEAT, NEAr-iron Transporter.

To monitor the conformational flexibility of linker-NEAT3 in the heme-free form, we employed HDX-MS. Samples of both heme-bound and heme-depleted linker-NEAT3 were separately subjected to deuterium exchange for 0.5, 1.0, 2.0, 4.0, 8.0, 16, 32, and 64 min, or for 0.5, 1.0, 2.0, 4.0, 8.0, 16, 32, 64, 128, and 256 min, and subsequently the data were analyzed with the HDExaminer software (Sierra Analytics) (Fig. 2, Tables S4 and S5). A control sample not subjected to deuterium exchange was also examined. The extent and rate of deuterium exchange provide useful information about the conformational differences between heme-bound and heme-free forms. Disordered residues and regions on the surface of the protein will have their amide hydrogens more exposed to the solvent, and in those cases, the degree of HDX will be greater than that in the regions that are well folded or buried in the core of the protein. In contrast, rigid regions will have amide hydrogens engaging in stable intramolecular hydrogen bonds, slowing down their deuterium uptake rate over time, whereas dynamic and flexible regions will exchange deuterium at a faster rate. Additionally, conformational changes conducive to slower HDX rate are generally an indicator of stabilization.

HDX-MS experiments were performed for up to 64 min or for up to 256 min. Although both sets of experiments showed similar trends, the experiment with shorter deuterium exchange incubation displayed a greater peptide coverage (Tables S4 and S5), and its results are displayed in Figure 2. Similar trends were observed in the second experiment lasting up to 256 min (Fig. S3). HDX-MS data revealed four regions experiencing a greater exchange of deuterium in the heme-free form than in the heme-bound form, suggesting that these regions are more flexible and/or more exposed in the absence of heme. These four regions comprised residues Lys587-Leu597 (region 1), Ala524-Phe534 (region 2), Ile632-Gln653 (region 3), and Ser557-Gly567 (region 4) (Fig. 2). Regions 1 and 2 belong to NEAT3 and linker domains, respectively. Interestingly, these two regions are located at the liker-NEAT3 interface, thus interacting with each other (Fig. 2*A*). At the start of the HDX-MS experiment, the extent of deuterium exchange between the heme-bound and the heme-free samples was similar. However, the level of deuteration increased faster in the heme-free from than in the heme-bound form. In other words, the deuteration exchange rates are greater in the absence of heme, indicating that these regions are more dynamic and flexible than in the presence of heme. In contrast, a higher level of deuterium uptake was observed in regions 3 and 4 in the heme-free than in the heme-bound forms from the beginning of the experiment, with little increase happening afterward. This result suggested that these regions are structured (low dynamics) and that binding of heme decreased the fraction of surface area accessible to the solvent. The structures of IsdH linker-NEAT3 bound to Hb and bound to heme are very similar, with an RMSD of 0.90 ± 0.06 Å. Therefore, we expect the identified regions in the HDX-MS will remain very similar in both structures with respect to heme and Hb binding.

Overall, the binding of heme resulted in smaller rates of deuterium uptake in two key regions in which the linker and NEAT3 interact with each other, suggesting a coupling between heme binding and stabilization of the linker-NEAT3 (locked conformation).

### Thermostability

**Figure 3:**
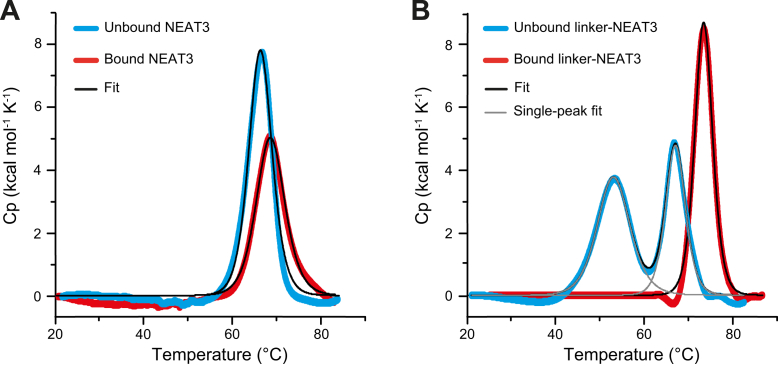
**Thermostability of IsdH constructs.***A and B*, DSC thermograms of (A) NEAT3 and (B) linker-NEAT3. Data for heme-free and heme-bound forms are shown in *blue* and *red* traces, respectively. The *black* and *gray* traces correspond to the overall fitting and the single-peak fittings (only in the sample of linker-NEAT3 without heme), respectively. Scans between 20 and 90 °C were performed in PBS at a protein concentration of 100 μM. DSC, differential scanning calorimetry; NEAT, NEAr-iron Transporter.

The loss of flexibility of linker-NEAT3 upon heme binding could have a significant impact on key physicochemical properties, such as thermal stability. The stability of linker-NEAT3 and NEAT3, each in the bound and heme-free forms, was evaluated by the high-resolution technique of differential scanning calorimetry (DSC) (Fig. 3 and Table S6). The transition midpoint (so-called melting temperature or *T*_*M*_) for the NEAT3 without heme bound was 65.6 °C (Fig. 3*A*). This temperature moderately increased to 67.8 °C in the presence of heme. This shift indicated a moderate stabilization of the NEAT3 domain upon heme binding. In the case of linker-NEAT3 with heme bound, the *T*_*M*_ was 73.2 °C (Fig. 3*B*), a value significantly greater (Δ*T*_*M*_ = 6.4 °C) than that of NEAT3 with heme bound. The enthalpy of the linker-NEAT3 with heme bound was also greater than that with NEAT3 (Table S6). Therefore, the linker domain had a stabilizing effect in the heme-bound form.

In the absence of heme, the linker-NEAT3 showed a characteristic unfolding pattern with two separate transition midpoints, one at 52.7 °C and a second one at 66.8 °C. The similarity of the second transition to the unfolding profile of NEAT3 suggested that the second peak in the linker-NEAT3 construct corresponds to the NEAT3 domain. The peak at a lower temperature was attributed to the linker region, and its distinct position indicated that the cooperative unfolding of the linker occurred separately from that of the NEAT3 region. Collectively, binding of heme to linker-NEAT3 stabilized both domains, leading to a single unfolding unit highlighting the relevant role of the linker domain in the stability of the heme-bound form of the protein.

### Heme binding

**Figure 4:**
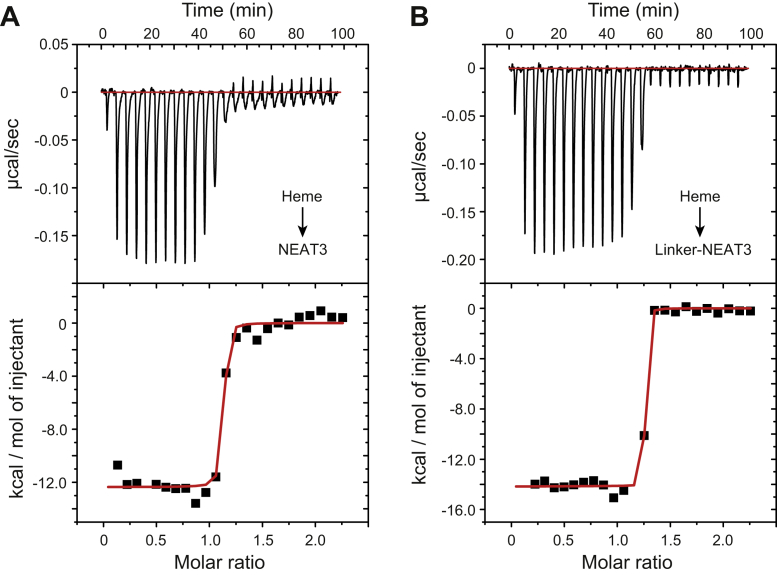
**Binding of heme to IsdH determined by ITC.***A*, binding of heme to NEAT3. *B*, binding of heme to linker-NEAT3. The *top panels* correspond to the titration kinetics, whereas the bottom panels represent the integrated binding isotherms. The experiments were carried out in PBS at pH 7.4 supplemented with 5% DMSO at a concentration of protein and heme of 4 and 50 μM, respectively. Molar ratio refers to the relative concentration (molar ratio) of heme with respect to protein throughout the titration. The binding enthalpy (*ΔH*) and the dissociation constant (*K*_*D*_) were determined by nonlinear regression of the integrated data to a one-site binding model with the program Origin. The values of *k*_on_ and *k*_*off*_ were determined from the titration kinetics (top portion of each panel) with the software AFFINImeter (50). All parameters are given in Table 2. DMSO, dimethyl sulfoxide; ITC, isothermal titration calorimetry; NEAT, NEAr-iron Transporter.

To evaluate the impact of the linker in heme binding, we employed the technique of isothermal titration calorimetry (ITC) (Fig. 4 and Table 2). The dissociation constant (*K*_*D*_) corresponding to the binding of heme to NEAT3 was 1.05 ± 0.15 nM. Binding was driven by a favorable enthalpy change (*ΔH* = −12.4 ± 2.5 kcal/mol). Similarly, the binding of heme to linker-NEAT3 was driven by a favorable enthalpy change (*ΔH* = −14.5 ± 1.1 kcal/mol) and showed high affinity, 0.51 ± 0.16 nM. Compared with NEAT3, the binding parameters of heme for linker-NEAT3 were very similar to each other, with only slightly stronger affinity for the latter.

**Table 2:** Thermodynamic and kinetic parameters corresponding to the binding of heme to IsdH

Construct	*K*_*D*_ (nM)	*ΔH* (kcal mol^−1^)	*-TΔS* (kcal mol^−1^)	*ΔG* (kcal mol^−1^)	*k*_*on*_ (M^−1^s^−1^) × 10^−6^	*N*	*k*_*off*_ (s^−1^) × 10^3^
**NEAT3**	1.05 ± 0.15	−12.4 ± 2.5	0.1 ± 0.3	−12.2 ± 0.1	3.9 ± 1.99	1.23 ± 0.01	3.8 ± 1.93
**Linker-NEAT3**	0.51 ± 0.16	−14.5 ± 1.1	1.9 ± 1.4	−12.7 ± 0.2	1.15 ± 0.25	1.18 ± 0.01	1.1 ± 0.23

The association rate constant (*k*_*on*_) and the dissociation rate constant (*k*_*off*_) were also determined from the ITC data (Table 2). The dissociation rate of heme from IsdB was reported to be 1.3 × 10^−3^ s^−1^ (31), and that of IsdA 2.6 × 10^−4^ s^−1^ (32). Our data (1.1 × 10^−3^ s^−1^ for linker-NEAT3 and 3.8 × 10^−3^ s^−1^ for NEAT3) are not so different from those in IsdB and IsdA. Since IsdH and IsdB transfer the heme moiety to IsdA, it is expected to see a slower heme dissociation rate from IsdA. The values indicate that both the association and dissociation steps are noticeably slower in the construct containing the linker. The structural/dynamic coupling of the linker domain with the NEAT3 domain upon heme binding could explain the slowdown of both the binding and the unbinding processes.

### Heme transfer experiments between NEAT3 domains.

**Figure 5:**
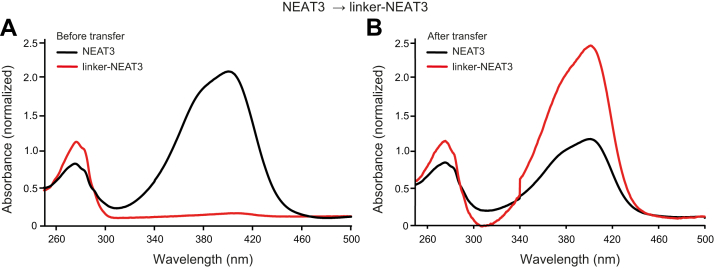
**Heme transfer from linker-NEAT3 to NEAT3.***A and B*, absorbance spectra of IsdH constructs (A) before the transfer and (B) after the transfer. The spectra of NEAT3 and linker-NEAT3 are shown with *black* and *red* traces, respectively. Absorbance was normalized to acquire a comparable spectrum of the Soret region (heme region). Details of the experiment are described in Experimental procedures. NEAT, NEAr-iron Transporter.

Qualitative heme transfer experiments from the linker-NEAT3 to heme-depleted NEAT3 (and vice versa) were carried out to evaluate the influence of the linker in heme transfer (Figs. 5 and S4). Previous research has shown that NEAT domains transfer the heme moiety not only to the NEAT domain of another protein of the Isd system but also to another NEAT domain of the same type of protein (*e.g.*, from the NEAT3 domain of one molecule of IsdH to a NEAT3 domain of another IsdH molecule) (33). The transfer of heme from IsdH to another NEAT domain causes a decrease of the Soret band at 405 nm that is correlated with the fraction of heme that has been transferred. In our experiment, we mixed heme-loaded linker NEAT3 with heme-depleted NEAT3, followed by their separation by a chromatographic technique as described in the Experimental procedures section. As a result of the experiment, the intensity of the Soret band in the sample containing linker-NEAT3 decreased, which was correlated with a concomitant increase of the absorbance in the sample containing NEAT3 (Fig. 5).

Similar results were observed when the experiment was conducted in the opposite direction, *i.e.*, the mixture was initially composed of heme-bound NEAT3 and heme-free linker-NEAT3 (Fig. S4). We note that in both sets of experiments, the Soret absorbance corresponding to the sample containing linker-NEAT3 was moderately greater than that of the sample containing only NEAT3. This observation indicated that a greater amount of heme is retained by linker-NEAT3 than by the NEAT3. This difference seemed to suggest a slightly higher affinity of heme for linker-NEAT3 than for NEAT3, an observation that was consistent with the ITC data shown earlier (Table 2).

### Heme acquisition from recombinant Hb

**Figure 6:**
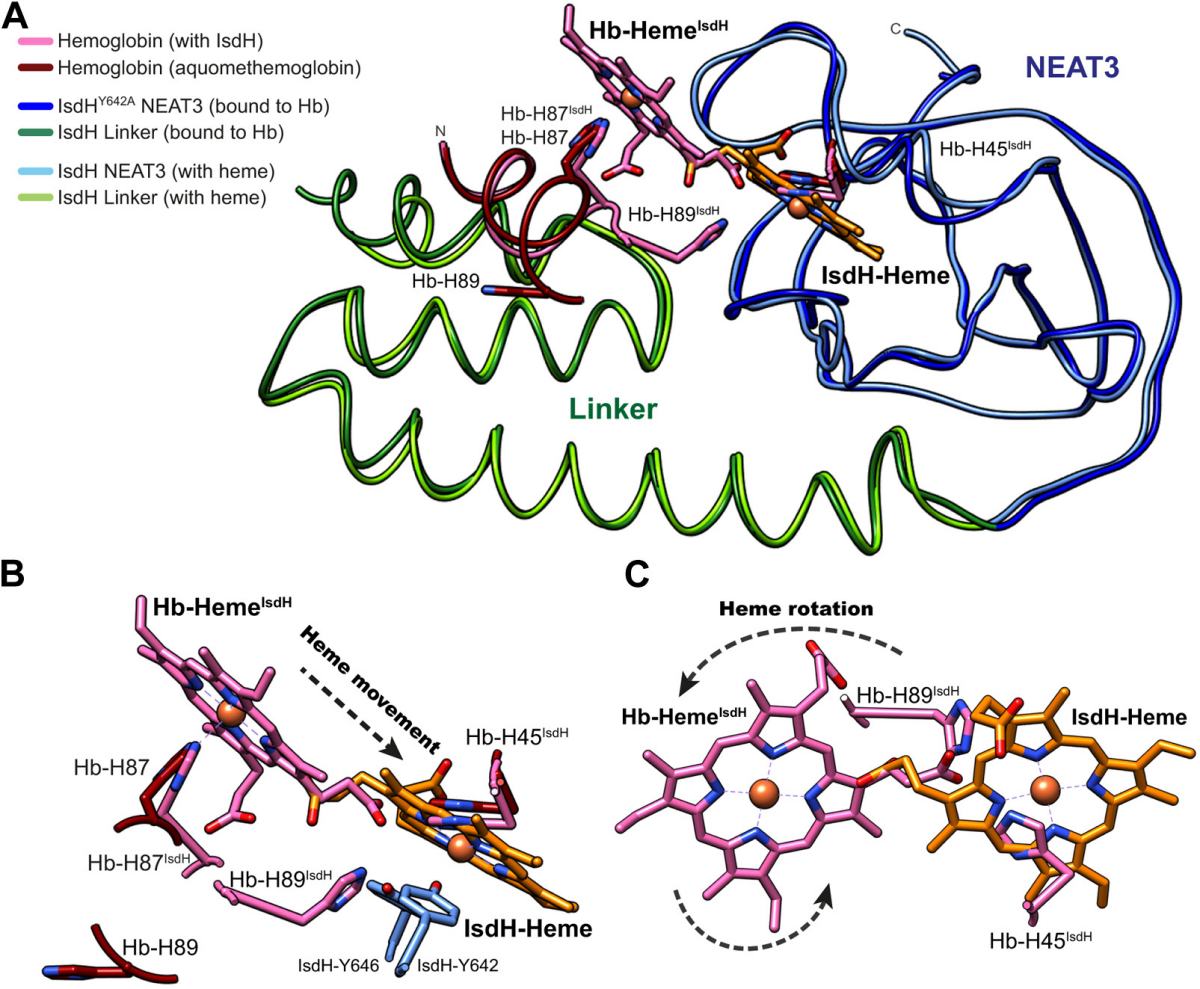
**Comparison of structures and heme movement.***A*, superposition of the crystal structure of human Hb (PDB entry code 3P5Q) (54), human Hb in complex with NEAT2-linker-NEAT3^Y642A^ (PDB entry code 4XS0) (19), and linker-NEAT3 in complex with heme (this work). Human Hb without IsdH bound is represented with *brown* ribbons, whereas human Hb in complex with IsdH is shown in *pink*. The NEAT2 domain is not shown. The linker and NEAT3^Y642A^ domains of the protein bound to Hb are shown in *dark green* and *dark blue*, respectively. Linker and NEAT3 in complex with heme are depicted in *light green* and *light blue*, respectively. The heme moiety bound to Hb in the structure in complex with IsdH is depicted in pink. The heme moiety bound to linker-NEAT3 is depicted with *orange sticks*. The iron atom is depicted with a sphere. Some key residues and the heme moieties are labeled. *B*, close-up view of the heme-binding region showing the movement of heme (indicated by the *dashed arrow*) to reach the binding pocket of IsdH. *C*, close-up view of the rotation of heme necessary to accommodate within the binding pocket of IsdH. The figure was prepared with UCSF Chimera (53). NEAT, NEAr-iron Transporter.

The linker has an important role in the interaction with Hb during heme acquisition (19, 20, 23, 24, 28). In particular, it has been reported that the linker is essential for the distortion of the F-helix of Hb, debilitating the affinity of Hb for heme (19, 28). In addition, previous studies have suggested that two histidine residues from Hb (His89 and His45) could participate in the movement of heme from Hb to NEAT3 (19, 24). This idea is based on the position of these two residues along the expected trajectory of heme when transferred from Hb to NEAT3. During the transfer step, heme must not only move from Hb to NEAT3 but also require a rotation to correctly dock into the binding pocket of NEAT3 (Fig. 6). To critically evaluate this hypothesis, we determined the relevant kinetic constants of heme transfer from a recombinant version of Hb to heme-free full-length IsdH, including mutations of His89 and His45 of recombinant Hb (rHb).

**Figure 7:**
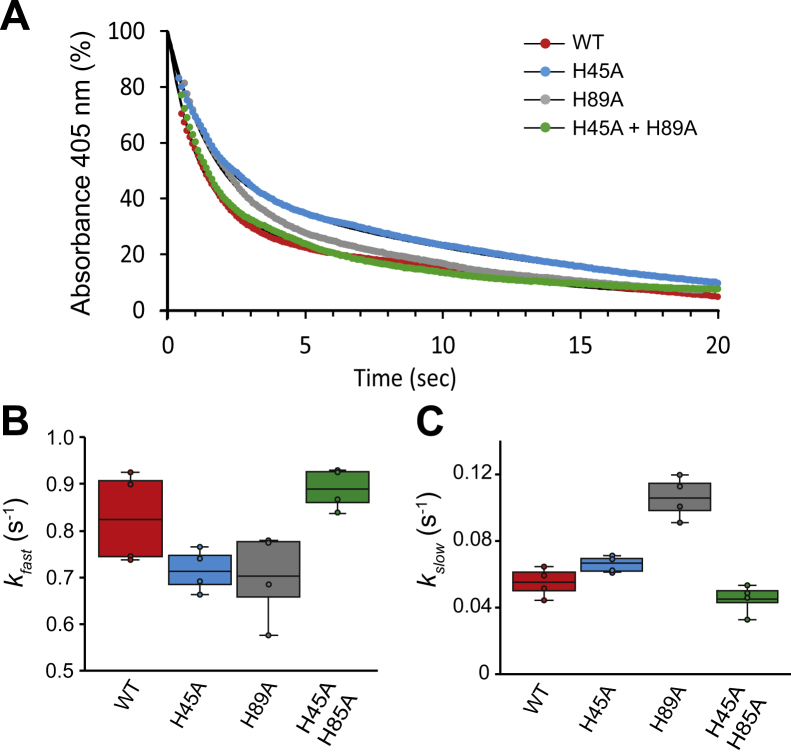
**Time course of heme acquisition from Hb.***A*, normalized UV/Vis absorbance at 405 nm collected over 20 s after mixing of different samples of rHb and heme-free NEAT1-NEAT3 in PBS with 0.5 M sucrose. The colored dotted plots corresponded to the normalized mean of absorbance of four independent experiments. The data colored in *red*, *blue*, *gray*, and green correspond to the values obtained for rHb WT, the single mutant H45A, the single mutant H89A, and the double mutant H45A/H89A, respectively. The black traces represent the fitting using a two-phase decay function. *B*, representation of *k*_*fast*_. *C*, representation of *k*_*slow*_. The middle line in each box represents the average of the independent values, which are represented by the dots. The *upper* and *lower* error bars represent the values of the standard deviation normalization and fitting were performed using the GraphPad Prism software. Other experimental details are as described in Experimental procedures. NEAT, NEAr-iron Transporter.

Heme transfer was monitored by the change of absorbance at 405 nm corresponding to heme and the kinetic parameters determined by fitting to a two-phase decay function (23, 24, 31) (Fig. 7 and Table S7). From these studies, the parameter *k*_*fast*_ was attributed to the heme transfer occurring upon interaction between Hb and IsdH, whereas *k*_*slow*_ was related to the dissociation of heme from Hb before acquisition by IsdH. We found no significant differences on the values of *k*_*fast*_ between WT rHb and its mutants, indicating that the mutation of the residues H45 and H89 did not greatly affect heme transfer from Hb to IsdH (Fig. 7B and Table S7). Regarding *k*_*slow*_, the mutant H89A showed an increased transfer rate, suggesting that this mutation may have partially destabilized the F-helix, thus increasing the spontaneous release of heme. Such change was not observed in the double mutant H89A/H45A, suggesting a compensation effect in the double mutant. Collectively, these data did not support the idea that His45 and His89 play a critical role in the rate-determining step of transfer of heme from Hb to full-length IsdH.

## Discussion

**Figure 8:**
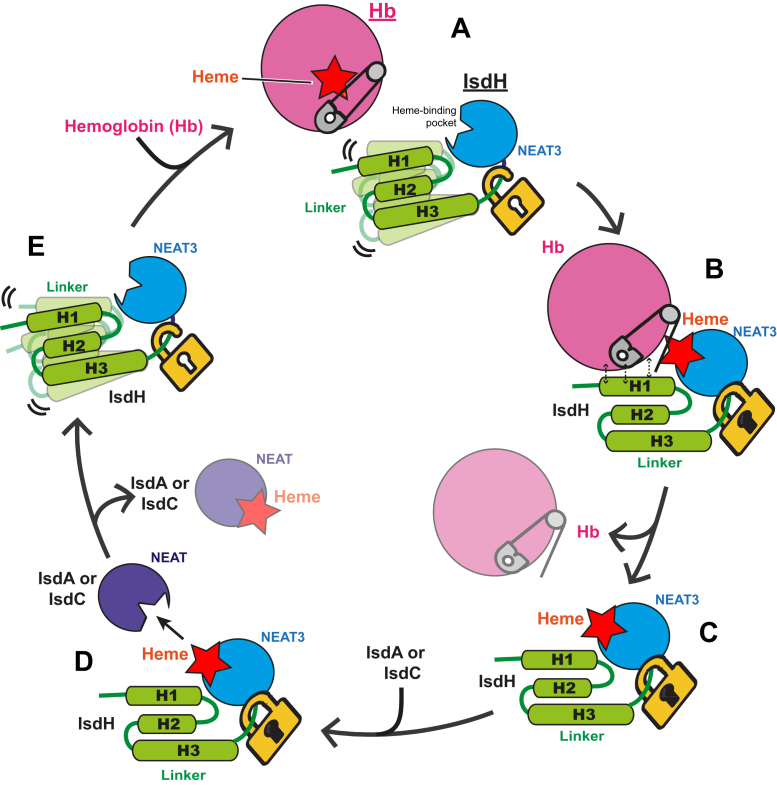
**Model of heme acquisition of IsdH from Hb.** Hb is represented with a *pink circle*, heme with a *red* star, NEAT3 with an indented *blue circle*, and the linker with three *green rectangles*. For clarity purposes the domain NEAT1-2 responsible for binding to Hb is not shown. *A*, Hb and linker-NEAT3 before binding. The F-helix of hemoglobin is represented as a safety pin and the linker flexibility indicated with the open padlock and the multiple conformations. *B*, upon binding, the linker and NEAT3 destabilize the F-helix of Hb (represented by the safety pin in the open conformation), which weakens heme binding to Hb and facilitates its movement to IsdH NEAT3. The loss of flexibility of the linker was represented with the locked padlock and a single conformation of the linker. *C*, after heme is acquired, IsdH releases the Hb moiety. The linker in the heme-bound form remains in a locked conformation. *D*, IsdH with heme bound interacts with IsdC or IsdA, and heme is transferred. *E*, as a result of heme transfer to IsdC or IsdA, the linker recovers its flexibility and gets ready to bind another molecule of Hb. NEAT, NEAr-iron Transporter.

The results obtained in this study have helped to gain a deeper insight into the mechanism of heme acquisition of IsdH, summarized in Figure 8. Specifically, the linker was shown to have a significant contribution to the stability of the heme-bound linker-NEAT3. The linker also contains a flexible region in the heme-free form, but does not significantly contribute to heme binding. *In vivo*, the flexibility of the linker region would facilitate the positioning of the NEAT3 domain when binding to Hb, helping to align the heme-binding pocket of IsdH NEAT3 with that of Hb. This flexibility would also facilitate the adjustment of IsdH to the interaction interfaces with the alpha- or with the beta-chain of Hb or the presence of haptoglobin in complex with Hb (23, 26). After heme binding to IsdH, the linker region will remain in a locked conformation to minimize interfering with the subsequent heme transfer from IsdH to the next Isd protein within the cell wall IsdA or IsdC (Figs. 2 and 3) (17, 25, 32). After heme is transferred from IsdH to IsdA or IsdC, the linker would regain its flexibility in order to initiate a new cycle of Hb binding and heme extraction (Fig. 8).

The relevance of the linker region is not limited to IsdH, but it is also present in other Isd-related proteins, such as IsdB from *S. aureus* (26), Shr from *Streptococcus pyogenes* (34), or IsdJ, IsdC, and IsdK from *Bacillus anthracis* (8, 35, 36, 37). It has been reported that the linker in *S. aureus* IsdB and in Shr from *S. pyogenes* are important in the interaction with Hb (15, 26, 29, 34). It is possible that the relevance of the linker flexibility for orientation and positioning described in this paper is a shared characteristic in other proteins involved in heme acquisition.

We have also shown that the heme must necessarily undergo a rotation when transferred from Hb to the binding pocket of NEAT3 (Fig. 6). A similar process has been reported during heme extraction by IsdB based on the crystal structure of the complex between Hb and NEAT1-linker-NEAT2 (15, 26). The heme rotates ∼90° from the Hb heme pocket to the IsdB heme pocket. When analyzing the heme transfer mechanism, it was observed that His45 and His89 of Hb are placed in the heme transfer route, and therefore, it has been hypothesized that these residues of Hb may favorably contribute to the effective transfer of the ligand. For example, His89 takes a position between the heme-binding pockets of Hb and IsdH, resulting from the distortion of the F-helix of Hb caused by the binding of IsdH to Hb. Heme transfer was measured using standard methods employed previously (24, 26, 28, 31). Based on the meager effect that mutating these residues to alanine had on the rate of heme transfer, we concluded that these residues do not participate in the rate-determining step of heme transfer (Fig. 7). The same Hb residues also appear in the heme transfer pathway from Hb to the NEAT2 domain of IsdB, resulting in a similar distortion of the F-helix of Hb (26). From our data and the structural similarities between IsdH and IsdB (15, 26, 29), it is possible to infer that these residues do not contribute significantly to the rate-determining step of heme transfer from Hb to IsdB, although it will be necessary to experimentally verify this hypothesis.

Collectively, the findings described herein contribute to our understanding of the heme acquisition mechanism by IsdH from Hb. We expect the conclusions of our research will help to clarify the mechanism of other Isd surface proteins such as IsdB and proteins of the IsdX system from *B. anthracis* (8, 35, 36, 37). Because NEAT domains are also reported to bind a number of host proteins other than Hb (among them fetuin, asialofetuin, fibrinogen, fibronectin, loricrin, involucrin, cytokeratin K10, and vitronectin), our results might be useful in subsequent studies characterizing these novel binding proteins at the molecular level (38).

## Experimental procedures

### Cloning, expression, and purification of IsdH constructs

IsdH full length was cloned into the expression vector pET28b by HiFi assembly method (New England Biolabs) using genomic DNA from *S. aureus* M50 as a template (12). An N-terminal His6-SUMO tag was included in the expression vector. The coding sequence of linker-NEAT3 was obtained by amplifying the coding region between Asn-476 and Gln-665 flanked by BamH1 and XhoI restriction sites, respectively, using a KOD-Plus mutagenesis kit (Toyobo) and subsequently cloned in a pET28-His_6_-SUMO vector. The expression vector for the NEAT3 domain comprising residues Pro539 to Gln664 with an N-terminal His_6_ tag and a thrombin cleavage site was obtained from a previous study (14).

### Expression of IsdH constructs

*Escherichia coli* C43 (DE3) competent cells were separately transformed with the expression vectors of the aforementioned IsdH constructs. Expression of heme-depleted protein was carried out in M9 minimal medium supplemented with kanamycin (5 mg L^−1^) as previously described (14). To produce the heme-bound form of IsdH (except for the thermostability assays, and the heme transfer experiments between NEAT domains), *E. coli* C43 (DE3) cells were transformed with the corresponding vector. Bacterial growth was carried out in LB medium supplemented with 100 μM FeCl_3_ (Wako) at 37 °C. When the absorbance at 600 nm reached a value of 0.4, expression of IsdH was induced by the addition of 0.5 mM isopropyl-β-D-thiogalactopyranoside (IPTG) and kept for 20 h at 28 °C under vigorous shaking.

### Purification of IsdH constructs

Cells were harvested by centrifugation at 4 °C for 10 min at 8000*g* (Tomy) and then resuspended in phosphate buffer saline (PBS, composed of 10.1 mM Na_2_HPO_4_·12 H_2_O, 1.8 mM KH_2_PO_4_, 2.7 mM KCl, 137 mM NaCl at pH 7.4) supplemented with 5 mM imidazole (Wako). The suspension of cells was lysed with an ultrasonic sonicator on ice for 10 min with 0.5 s pulses (Tomy). The soluble fraction was separated by centrifugation for 30 min at 20,000 rpm (Tomy) at 4 °C, filtrated with a 0.22-μm sterile filter (Millex GP) and loaded into a gravity open column to perform immobilized-metal affinity chromatography (IMAC) using Ni-NTA agarose (Qiagen). The column was equilibrated with PBS containing 5 mM imidazole prior to loading the soluble fraction. The column was washed with 10 column volumes of PBS containing 15 mM imidazole. Subsequently, the protein was eluted with a step gradient of imidazole from 15 to 500 mM in PBS buffer, and the fractions containing the protein were collected.

To remove SUMO-tag from IsdH and linker-NEAT3, protein samples were each incubated with UlpI protease at a mass ratio of 1:10 (Ulp1:IsdH protein) simultaneously to a dialysis against PBS for 20 h at 4 °C. When purifying the NEAT3 domain, the cleavage of the N-terminal His_6_ tag was carried out with thrombin (Sigma-Aldrich) for 20 h at 4 °C (1 U of thrombin per mg of protein) simultaneously to dialysis in PBS. In all cases, the cleaved proteins were separated from His_6_-SUMO tag and UlpI protease, or from His_6_-tag, by an additional IMAC using Ni-NTA agarose. We note that the protein thrombin is not separated by IMAC, but instead is separated during the size-exclusion chromatography step (see below). The open Ni-NTA agarose gravity column was first equilibrated with PBS containing 15 mM imidazole, and after loading the proteins, IsdH, linker-NEAT3, or NEAT3 was collected in the flow-through fraction or in the first washing fractions with PBS supplemented with 15 mM imidazole. We note that the His_6_-SUMO-tag attached to linker-NEAT3 was not removed when employed for heme transfer assays.

The last purification step for all IsdH constructs was size-exclusion chromatography using a HiLoad 16/600 superdex pg-200 column equilibrated with PBS buffer in an AKTA system (GE Healthcare). The fractions containing the target proteins were pooled together and used immediately or flash-frozen in liquid nitrogen and stored at −70 °C. Protein concentrations of full-length IsdH, linker-NEAT3, and NEAT3 were measured on NanodropOne (ThermoFisher Scientific) using molar extinction coefficients 89,620, 21,890 and 15,930 cm^−1^ M^−1^, respectively (39).

### Expression and purification of Ulp1

The gene encoding the protease UlpI with an N-terminal His_6_ tag cloned in a modified pET28 vector (GenBank KJ782405.1) was expressed in *E. coli* BL21(DE3). Protein expression was induced by the addition of IPTG at 1 mM, after which the cells were grown for an additional 20 h at 28 °C. The cells were harvested by centrifugation at 4 °C for 10 min at 8000*g* and subsequently resuspended in 20 mM Tris buffer, pH 7.4, containing 300 mM NaCl and 20 mM imidazole. Cells were disrupted by sonication on ice for 10 min with 0.5-s pulses. The soluble portion was obtained by centrifugation (20,000 rpm for 30 min at 4 °C) and applied to an IMAC gravity open column (Ni-NTA agarose) equilibrated with 20 mM Tris buffer at pH 7.4, containing 300 mM NaCl and 20 mM imidazole. After washing the column with 10 column volumes of the same buffer, UlpI was eluted with a step gradient of imidazole from 20 to 500 mM. The fractions containing UlpI were pooled together and subsequently loaded into a Superdex 16/600 pg-75 column in an AKTA system (GE Healthcare) equilibrated with 20 mM Tris buffer, pH 7.4, and containing 300 mM NaCl. The protein concentration was determined as aforementioned using a molar extinction coefficient of 30,098 cm^−1^ M^−1^ (39). The purified protein was flash-frozen with liquid nitrogen and stored at −70 °C until use.

### Expression and purification of recombinant human Hb and its mutants

The vector pHE2 containing the sequence of WT human α-globin and β-globin genes placed in tandem under a tac-promoter (40) was kindly provided by Dr Tjandra from the National Institutes of Health Bethesda (MA) (41). The single mutations of rHb H45A and H89A, and the double mutation H89A/H45A were introduced in the sequence of rHb by site-directed mutagenesis using a KOD-Plus mutagenesis kit (Toyobo). rHb expression and purification for wildtype (WT) and mutants were performed separately according to the methodology previously reported, with minor modifications (42). Briefly, transformed *E. coli* JM109 (DE3) cells were grown in terrific broth medium in a 3-L bioreactor (ABLE Biott, BMS-P) at 28 °C for 20 h. Expression was induced by adding 0.2 mM IPTG. The culture was supplemented with hemin chloride at 20 mg L^−1^ (Tokyo Chemical Industry) and 10 g L^−1^ glucose (Wako), and growth was continued for another 5 h at 20 °C. The cells were harvested by centrifugation at 4 °C for 10 min at 8000*g*, resuspended in 20 mM Tris HCl buffer at pH 7.4, and lysed with an ultrasonic cell disruptor (Tomy) for 8 minutes with 0.5-s pulses in an ice bath. Lysed cells were centrifuged at 4 °C for 30 min at 20,000 rpm (Tomy). The supernatant was filtered with a 0.22-μm sterile filter (Millex GP) and saturated with CO gas (GL Sciences) for 10 min on ice.

Three chromatographic steps in an AKTA system (GE Healthcare) were employed in the purification process. First, the soluble cell lysate was subjected to ion exchange chromatography in a 20-mL HiPrep Q-Sepharose 16/60 Q XL column (Cytiva) with 20 mM Tris HCl at pH 7.4. Most unwanted protein binds to the resin, while rHb appears in the flow-through fraction. This solution was dialyzed against 20 mM Tris HCl at pH 8.3 and subsequently subjected to a second ion-exchange step in a 5-mL HiTrap SP QHP column (Cytiva) with a NaCl gradient from 0 to 160 mM. Recombinant Hb eluted in one major peak. The resultant protein was dialyzed against 10 mM sodium phosphate at pH 6.8 prior to the third ion-exchange chromatography, for which a 1-mL Resource S column (GE Healthcare) was employed. The protein was eluted with a pH gradient using 10 mM sodium phosphate (pH 6.8) and 20 mM sodium phosphate (pH 8.3). The protein rHb (WT and mutants) was obtained in one main peak, with high purity as judged by SDS-PAGE. The concentration of rHb (31.2 kDa) was determined by bicinchoninic acid protein assay kit using bovine serum albumin as standard (ThermoFisher Scientific). The absorbance at 562 nm was measured in a plate-reader EnSpire instrument (PerkinElmer).

### Purification of linker-NEAT3 for crystallization

The gene encoding full-length IsdH with an N-terminal His_6_ tag in pET28b was expressed in *E. coli* Rosetta2 (DE3). After inducing its expression with IPTG, the cells were grown for four additional hours. Harvested cells were resuspended in PBS buffer supplemented with 20 mM imidazole and disrupted by the method of sonication. The soluble portion was obtained by centrifugation (40,000*g*, 30 min) and applied to an IMAC column (Ni^2+^-NTA). The protein was eluted with an imidazole gradient (20–500 mM). The fractions containing protein were pooled together, followed by treatment with thrombin (1 U/mg protein) overnight. The sample was subsequently subjected to a second IMAC in which the flow-through fractions were pooled together and subsequently subjected to size-exclusion chromatography in a column equilibrated with 50 mM phosphate at pH 7.4. During the purification, the pure protein fraction obtained did not correspond to full-length protein, but to linker-NEAT3 as determined from the structural data (see Crystallization, data collection and refinement section).

### Crystallization, data collection, and refinement

The linker-NEAT3 protein obtained from above at 10 mg ml^−1^ was crystallized in a solution composed of 0.2 M potassium chloride and 20% PEG 3350 (Hampton Research) at 298 K. Suitable crystals were harvested, briefly incubated in mother liquor supplemented with 20% glycerol, and transferred to liquid nitrogen for storage until data collection. Diffraction data from a single crystal of IsdH-linker-NEAT3 were collected in beamline BL5A at the Photon Factory (Tsukuba) under cryogenic conditions (100 K). Diffraction images were processed with the program MOSFLM and merged and scaled with the program SCALA (43) of the CCP4 suite (44). The structure of the protein was determined by the molecular replacement method using the coordinates of NEAT3 (PDB entry code 3VTM) (14) protein from above with the program PHASER (45). The model was refined with the programs REFMAC5 (46) and built manually with COOT (47). Validation was carried out with PROCHECK (48). Data collection and structure refinement statistics are given in Table 1.

### N-terminal sequencing

We attempted to crystallize the linker-NEAT3 in the heme-free form (purified from the His_6_-SUMO-linker-NEAT3 construct), but the numerous crystals obtained only contained NEAT3, indicating that the linker domain was cleaved during the crystallization period of weeks. To determine the position of the cleavage site, we first subjected the crystallization samples to SDS-PAGE. The protein bands were transferred to a polyvinylidene fluoride membrane (Bio-Rad) and stained with Ponceau 3R solution (Wako) for 1 h to verify that the transfer was successful. Excess dye was removed by successive rinses with water. The bands of protein were excised from the membrane, and their N-termini were sequenced by standard methodologies (49).

### HDX-MS

The linker-NEAT3 at 1 mg/ml with or without heme was diluted 10-fold with PBS in D_2_O. The diluted solutions were separately incubated at 10 °C for 0.5, 1.0, 2.0, 4.0, 8.0, 16, 32, and 64 min or for 0.5, 1.0, 2.0, 4.0, 8.0, 16, 32, 64, 128, and 256 min. Deuterium-labeled samples were quenched by adjusting to pH 3.0 with equal volumes of quenching buffer (0 °C) containing 1 M TCEP-HCl, (Sigma-Aldrich) and 2 M Urea (Wako) using HDx-3 PAL (LEAP Technologies). All time points were determined with three independent labeling experiments. After quenching, the solutions were subjected to online pepsin digestion and analyzed by LC/MS using UltiMate3000RSLC nano-connected to the Q Exactive Plus mass spectrometer (both ThermoFisher Scientific). Online pepsin digestion was performed using a Poroszyme Immobilized Pepsin Cartridge (2.1 × 30 mm, ThermoFisher Scientific) in formic acid solution at pH 2.5 for 3 min at 8 °C and at a flow rate of 50 μl/min. Acclaim PepMap300 C18 (1.0 × 15 mm, ThermoFisher Scientific) and Hypersil Gold columns (1.0 × 50 mm, ThermoFisher Scientific) were used as desalting and analytical columns. The mobile phase was 0.1% formic acid solution and 0.1% formic acid containing 90% acetonitrile. The deuterated peptides were eluted at a flow rate of 45 μl/min with a gradient of 10% to 90% acetonitrile buffer. The conditions of the mass spectrometer were settled in an electrospray voltage of 3.8 kV, positive-ion mode, sheath and auxiliary nitrogen flow rate at 20 and 2 arbitrary units, ion transfer tube temperature at 275 °C, auxiliary gas heater temperature at 100 °C, and mass range of 200 m/z to 2000 m/z. Data-dependent acquisition was performed with normalized collision energy of 27 arbitrary units. The MS and MS/MS spectra were subjected to a database search analysis using the Proteome Discoverer 2.2 (ThermoFisher Scientific) against an in-house database containing the amino acid sequence of the protein. The search results and MS raw files were used for the analysis of the deuteration levels of the peptide fragments using the HDExaminer software (Sierra Analytics), and the data obtained were represented using the crystal structure of bound IsdH linker-NEAT3.

### Thermostability

Thermostability was monitored by DSC in a PEAQ-DSC instrument (MicroCal). The heme-bound form was obtained by addition of excess of hemin chloride at a molar ratio of 3:1 (heme:protein). First, 6 mM hemin chloride was dissolved in 100% dimethyl sulfoxide (DMSO) (Wako) and subsequently serially diluted in decreasing concentrations of DMSO (100% to 60%, followed by dilution to 20% and in the last step to 10% DMSO). The diluted heme was added to the protein, to a final concentration of 300 μM heme and 100 μM protein in 5% DMSO. Removal of hemin excess was carried out in a PD-10 desalting column equilibrated in PBS buffer following the instructions of the manufacturer (Cytiva). Proteins were dialyzed overnight at 4 °C in PBS (no DMSO present) and prepared to a final protein concentration of 100 μM. Measurements were performed at a scan rate of 1 °C per minute from 20 °C to 90 °C. Filtered PBS buffer from the dialysis was used as the reference sample to obtain the baseline. The thermogram was evaluated after subtracting the baseline. The parameters of thermostability (melting temperature (*T*_*M*_), and enthalpy change (Δ*H*)) were determined with the software integrated in the instrument.

### Isothermal titration calorimetry

Binding of heme to IsdH constructs was monitored by ITC in a VP-ITC instrument (MicroCal). Proteins were dialyzed in PBS at 4 °C pH 7.4. The cell and syringe were filled with 4 μM protein (heme-free) and hemin chloride 50 μM, respectively. The buffer was PBS supplemented with 5% DMSO. An initial injection of hemin (0.5 μl) was followed by 23 injections of 10 μl every 240 s at 25 °C. The syringe was stirred at 750 rpm. The data were analyzed with the program ORIGIN (MicroCal) using a one-site binding model. The kinetic parameters *k*_*on*_ and *k*_*off*_ were calculated from the raw calorimetric data with the program AFFINImeter (50) based on the kinITC methodology (51, 52). In kinITC, the variation of the time needed to return to the baseline in each injection is plotted as a function of the stoichiometric ratio reached in each injection, from which *k*_on_ and *k*_off_ were then obtained.

### Heme transfer experiments between NEAT domains

Transfer experiments of heme between NEAT3 constructs were performed as previously described (33). Briefly, hemin chloride was first dissolved in 0.1 M NaOH (Wako) to a concentration of 1 mM and subsequently diluted in PBS to the desired concentration. The protein used as heme donor (the heme-bound form) was obtained by addition of the freshly prepared hemin chloride to the heme-free protein in PBS at a 3:1 M ratio (heme to protein). The excess heme was removed in a PD-10 desalting column in PBS buffer. Equal amounts of heme-bound IsdH linker-NEAT3 bearing an intact His6-SUMO tag was mixed with heme-free IsdH NEAT3 at 48 μM for 10 min. The protein mixture was separated by IMAC in a Ni-NTA agarose column equilibrated with PBS buffer. The fractions containing NEAT3 were collected in the flow-through in PBS buffer supplemented with 15 mM imidazole. The IsdH linker-NEAT3 protein bound to the column was treated with protease UlpI (cleaving the His6-SUMO tag) at 4 °C for 2 h. The linker-NEAT3 construct was then eluted with PBS supplemented with 15 mM imidazole. The UV-visible spectra between 250 and 500 nm of NEAT3 and linker-NEAT3 were recorded separately in a UV-Vis spectrophotometer (JASCO V-660) at 25 °C using a 10-mm quartz cell (12, 14, 33). For the transfer of heme from NEAT3 to linker-NEAT3, the former was treated with hemin to obtain the bound form and excess heme was removed, followed by the incubation with heme-free linker-NEAT3 as described earlier. The fractions of NEAT3 and linker-NEAT3 were collected and examined as explained for the reverse transfer experiment outlined earlier.

### Heme acquisition from Hb

Heme acquisition of IsdH full-length with rHb was monitored at 405 nm in a UV-Vis spectrophotometer (JASCO V-660) at 15 °C using a 10 mm quartz cell (23, 24, 26, 28, 31). To maintain pseudo-zero-order reaction conditions, the experiments were performed at a 1:20 stoichiometric ratio of rHb with respect to IsdH. The samples were prepared in a solution of PBS buffer supplemented with 0.5 M sucrose (Wako) as described earlier (23, 24). The freshly purified rHb samples (WT or alanine mutants) were equilibrated into the quartz cell in the UV-spectrophotometer. After IsdH was added, the transferring process was monitored for 20 s at 405 nm. Four independent replicates were performed for each variant of rHb. Fitting to a two-phase decay function (24) was performed using the GraphPad Prism 9 software.

## Data availability

The coordinates and structure factors of linker-NEAT3 with heme bound have been deposited in the Protein Data Bank with entry code 7W81. All remaining data are contained within the article.

## Conflict of interests

The authors declare no conflict of interest.
