# Supplementary material for: Structure and role of the linker domain of the iron surface-determinant protein IsdH in heme transportation in Staphylococcus aureus
Source: J Biol Chem. 2022 Apr 29;298(6):101995. doi: 10.1016/j.jbc.2022.101995 (PMC9163592; doi:10.1016/j.jbc.2022.101995)
Supplement: Supplemental Tables S1–S7 and Figures S1–S4 [file mmc1.pdf]

Supporting information

Structure, dynamics and mechanism of the heme transporter IsdH from *Staphylococcus aureus*: Role of the linker domain.

Sandra Valenciano-Bellido[1], Jose M. M. Caaveiro[1,2,*], Koldo Morante[1], Tatyana Sushko[1], Makoto Nakakido[1,3], Satoru Nagatoishi[4], and Kouhei Tsumoto[1,3,4,*]

[1]*Department of Bioengineering, School of Engineering, The University of Tokyo, 7-3-1 Hongo, Bunkyo-ku, Tokyo 113-8656, Japan;* [2]*Laboratory of Global Healthcare, Graduate School of Pharmaceutical Sciences, Kyushu University, 3-1-1 Maidashi, Higashi-ku, Fukuoka 812-8582, Japan;* [3]*Department of Chemistry and Biotechnology, Graduate School of Engineering, The University of Tokyo, 7-3-1 Hongo, Bunkyo-ku, Tokyo 113-8656, Japan;* [4]*Institute of Medical Science, The University of Tokyo, 4-6-1 Shirokanedai, Minato-ku, Tokyo 108-8639, Japan.*

* Corresponding author: Jose M.M. Caaveiro (jose@phar.kyushu-u.ac.jp)

* Corresponding author: Kouhei Tsumoto (tsumoto@bioeng.t.u-tokyo.ac.jp)

Table S1: Sequence of IsdH linker-NEAT3.

Residue number	Sequence of IsdH linker-NEAT3
**476-526**	NLQKLLAPYHKAKTLERQVYELEKLQEKLPEKYKAEYKKKLDQTRVELADQVKSA
**527-578**	VTEFENVTPTNEQLTDLQEAHFVVFESEENSESVMDGFVEHPFYTATLNGQKYVVM
**579-629**	KTKDDSYWKDLIVEGKRVTTVSKDPKNNSRTLIFPYIPDKAVYNAIVKVVVANIGYE
**630-655**	GQYHVRIINQDI

The N-terminal sequence of the freshly purified protein, or that of the crystallized protein is shown in blue and red, respectively

Table S2. Sequence of the N-terminal of freshly purified linker-NEAT3.

Residue number	Identity	Sequence number
1	Serine	Cloning artifact
2	Asparagine	477
3	Leucine	478
4	Glutamine	479
5	Lysine	480

Table S3. Sequence of the N-terminal of the linker-NEAT3 after crystallization.

Residue number	Identity	Sequence number
1	Alanine	526
2	Valine	527
3	Threonine	528
4	Glutamic Acid	529
5	Phenylalanine	530

Table S4. Summary of HDX-MS experiment[a].

Data Set	Linker-NEAT3	Heme + linker-NEAT3
Temperature (ºC)	10	10
HDX time course (s)	0, 30, 60, 120, 240, 480, 960, 1920, 3840	Same as in linker-NEAT3
Number of peptides	211	225
Sequence coverage (%)	98.9	98.9
Average peptide length	15.1	14.8
Redundancy	17.1	18.0
Replicates	1	1

[a] Table obtained from the data generated in the HDExaminer software (Sierra Analytics).

Table S5. HDX-MS experiment data summary for 256 minutes[a].

Data Set	Linker-NEAT3	Heme + linker-NEAT3
Temperature (ºC)	10	10
HDX time course (s)	0, 30, 60, 120, 240, 480, 960, 1920, 3840, 7680, 15360	Same as in linker-NEAT3
Number of peptides	131	135
Sequence coverage (%)	87	87

[a] Table obtained from the data generated in the HDExaminer software (Sierra Analytics).

Table S6. Thermostability parameters of IsdH constructs

**Construct**	**Tm 1 (ºC)**	**Tm 2 (ºC)**	***ΔH* 1 (kcal mol^{-1})**	***ΔH* 2 (kcal mol^{-1})**
NEAT3 heme-free	-	65.6 ± 0.1	-	53.7 ± 0.1
NEAT3 heme bound	-	67.8 ± 0.1	-	42.3 ± 0.1
Linker-NEAT3 heme free	52.7 ± 0.1	66.8 ± 0.1	37.2 ± 0.1	28.6 ± 0.1
Linker-NEAT3 heme bound	-	73.2 ± 0.1	-	46.8 ± 0.7

Table S7. Kinetic parameters of heme transfer.[a]

**Mutation**	k_{fast} (s^{-1})	k_{slow} (s^{-1})
WT	0.82 ± 0.1	0.054 ± 0.01
H45A	0.71 ± 0.05	0.065 ± 0.005
H89A	0.69 ± 0.1	0.105 ± 0.013
H45A / H89A	0.88 ± 0.04	0.043 ± 0.01

[a]Normalization, fitting and error (confidence factor 95%) was performed using GraphPad Prism.

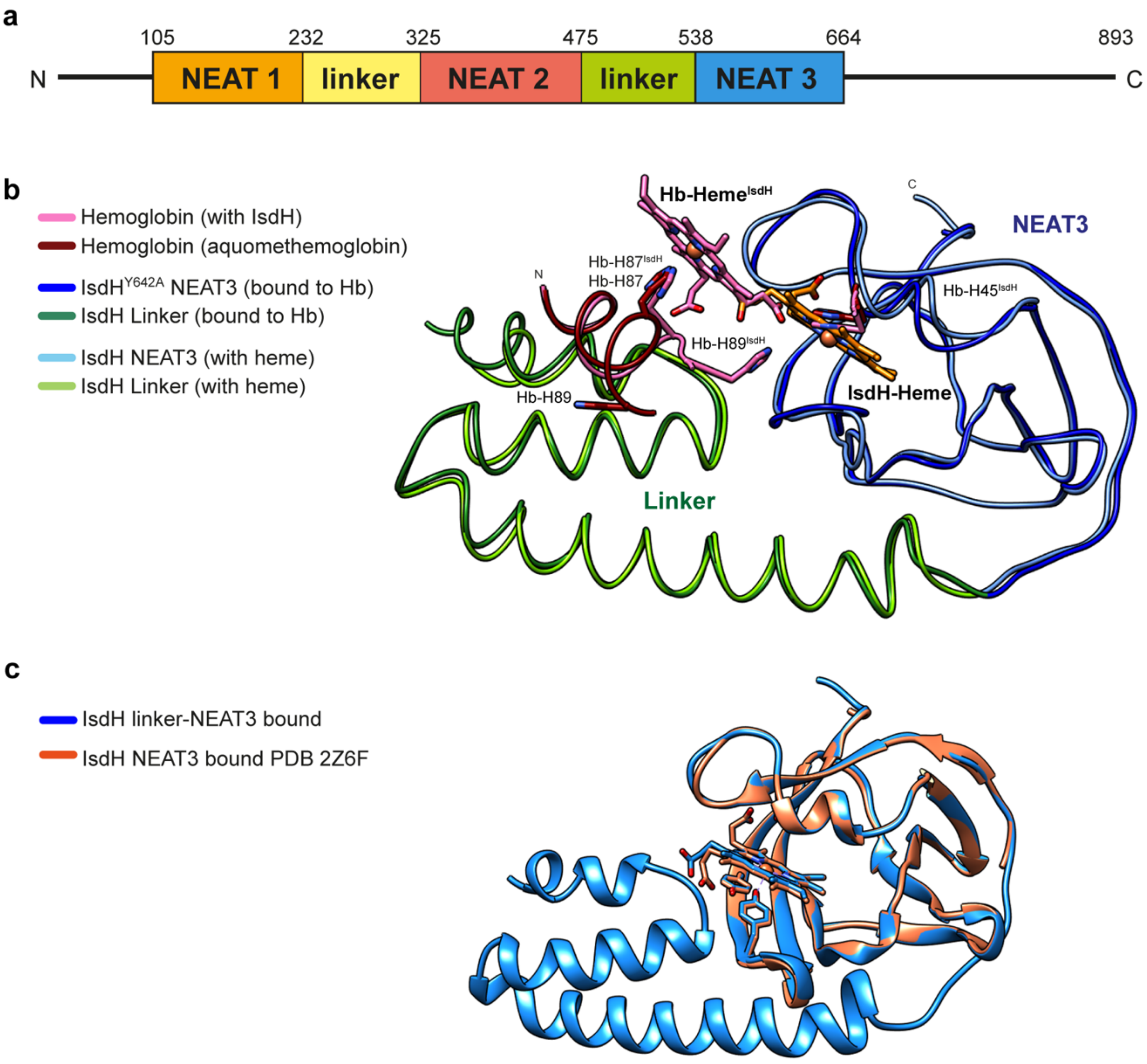

Figure S1. Comparison of structures. (a) Superposition of the crystal structure of human Hb (PDB entry code 3P5Q) (54), human Hb in complex with NEAT2-linker-NEAT3^{Y642A} (PDB entry code 4XS0) (19), and linker-NEAT3 with heme bound (this work). Human Hb free of IsdH is represented with brown ribbons, whereas human Hb in complex with IsdH is shown in pink. The NEAT2 domain is not shown. The linker and NEAT3^{Y642A} domains of the protein bound to Hb are shown in dark green and dark blue, respectively. The structure of the linker and NEAT3 with heme bound is depicted in light green and light blue, respectively. The heme moiety bound to Hb in the structure in complex with IsdH is depicted in pink. The heme moiety bound to linker-NEAT3 is depicted with orange sticks. The iron atom is depicted with a sphere. Some key residues and the heme moieties are labeled. **(c)** Superposition of the crystal structure of NEAT3 in complex with heme (depicted in orange, PDB entry code 2Z6F (14)) and the linker-NEAT3 in complex with heme (depicted in blue, this study). Only the residues corresponding to the NEAT3 domain were employed for the superposition. Panels (b) and (c) were prepared with UCSF Chimera.

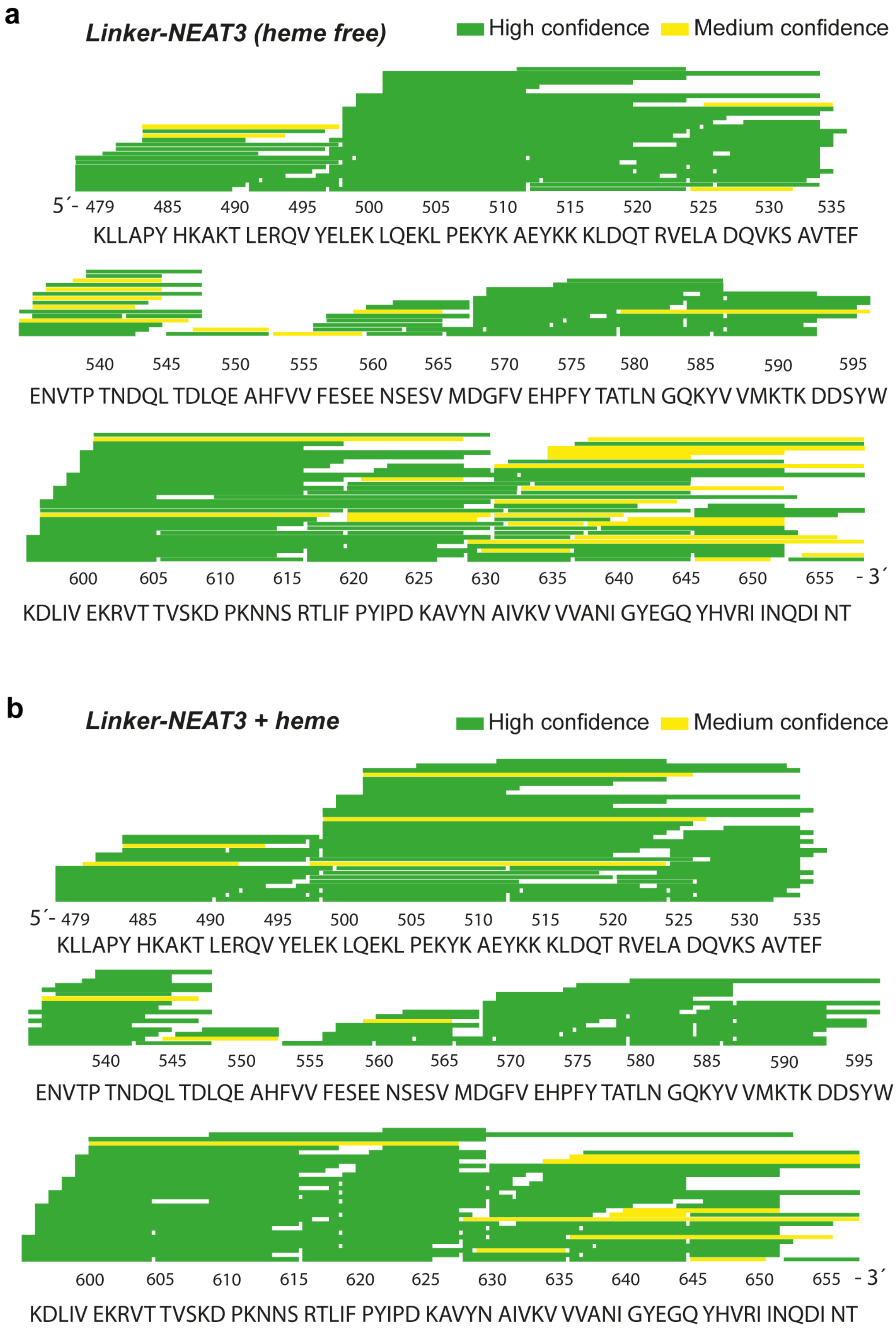

Figure S2. HDX-MS peptide coverage of linker-NEAT3. The linker-NEAT3 was mapped for the heme-free **(a)** and the heme-bound **(b)** forms. Peptides are plotted after a sequence identification run, represented as green or yellow bars corresponding to a high or medium confidence level, respectively, and aligned to their relative position in the sequence. Peptide coverage was obtained by using the HDExaminer software (Sierra Analytics).

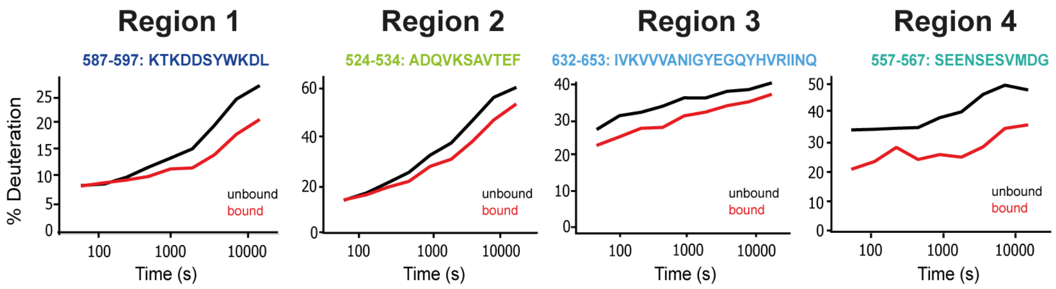

Figure S3. Time course of deuterium exchange for the four regions of interest of linker-NEAT3 (same as in Figure 2). The experiment corresponds to a second determination with longer incubation in deuterated medium. Each panel shows the degree of deuterium exchange for each segment (indicated at the top of each panel) in the heme-free (black line) and heme-bound (red line) forms.

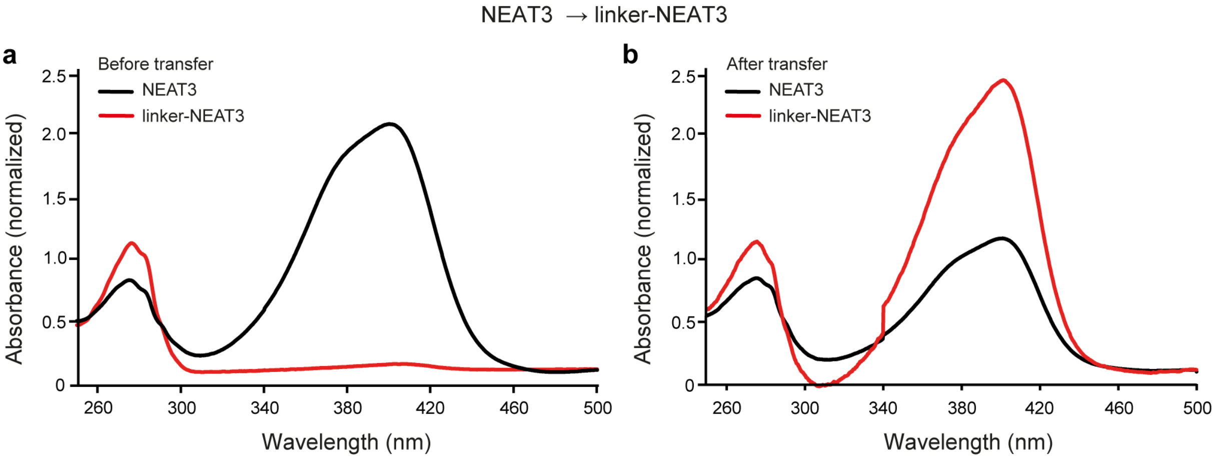

Figure S4. Heme transfer from NEAT3 to linker-NEAT3. Absorbance spectra of IsdH constructs **(a)** before the transfer and **(b)** after the transfer. The spectra of NEAT3 and linker-NEAT3 are shown with black and red traces, respectively. Absorbance was normalized to acquire a comparable spectrum of the Soret region. Details of the experiment are described in materials and methods.
